# Firms’ approach to mitigating risks in the platinum group metals sector

**DOI:** 10.1007/s13563-021-00249-4

**Published:** 2021-02-08

**Authors:** Gracelin Baskaran

**Affiliations:** 1 University of Cambridge, Allison Richards Building, 7 West Road, Cambridge, CB3 9DT UK; 2 Development Policy Research Unit, School of Economics, University of Cape Town, Rondebosch, 7701 South Africa

**Keywords:** Mining, Firm behavior, South Africa, Platinum group metals, Risks, Shocks, Commodities, Extractives

## Abstract

With platinum prices declining due to a seismic demand shift and firms grappling with the high cost of labor and prolonged production-stopping labor strikes, the financial sustainability of platinum group metal (PGM) firms has come under duress since 2012. This article seeks to understand how mining firms have reshaped their strategies to ensure financial sustainability while also meeting external expectations of increasing shared-value outcomes by assessing how firms are deploying buffers and bridges. Firms use buffers when they seek to protect core business activities from supply-side and demand-side volatilities by shifting their overall business strategy and bridges to conform with external expectations of improving shared-value outcomes. The article constructs a case study using five sets of data to assess (i) what supply and demand shocks firms are facing; (ii) how they are reshaping their strategies and undertaking activities to protect the firm from these shocks; (iii) what challenges firms face with reaching shared-value outcomes; and (iv) how firms are undertaking activities to improve shared-value outcomes. This article finds that although buffering activities have been successful at increasing the financial sustainability of mining activities, bridging activities have been less successful given that royalties are vulnerable to maladministration, the impact of corporate social investments often short-lived, and insular and local procurement is limited to low-value-added activities, particularly in the context of mechanization. This article seeks to contribute to the body of literature on how extractive firms are responding to supply and demand shocks and argues that a paradigm shift may be necessary in which traditional bridging activities become part of protecting core business activities to ameliorate the risk of losing a firm’s social license to operate.

## Introduction

### Background

Between 2002 and 2008, platinum prices increased by 354%. Given that South Africa owns 75% of the world’s platinum deposits, firms benefited immensely from this unprecedented rise. For example, Anglo Platinum’s earnings rose by 43% ($1.8 billion) between just 2004 and 2005. The majority of platinum mined during this time came from large industrial mines located on South Africa’s platinum belt, found in the North West Province near Rustenburg, Marikana, and Brits. It was an immensely profitable time for mining firms.^1^ The City of Rustenburg was labeled as the “fastest-growing city in Africa” after Cairo (Rajak 2012). However, by July 2009, the “boom” was starting to fade. Anglo Platinum announced a 69% decline in profits, to $1 billion for the first half of the year, and by the end of the year, retrenchments began to reduce operating costs (Rajak 2011).

Although the boom and bust cycle is not new to Rustenburg, South Africa, or mining towns across the world, this cycle could be permanently disrupted by technological changes to the automotive sector. Without many other high value-added uses for platinum, this decline has the potential to become secular rather than cyclical. In general, the mining sector globally is cyclical in nature, as varying supply and demand factors contribute to cyclical business conditions. A cyclical market is characterized by peak-trough-peak movements—often referred to “booms” and “busts” in mining. On the other hand, a secular market is a long-term event with persistent conditions irrespective of economic slowdowns and cycles. One such example of a secular market is the tin sector in Cornwall. An analysis by Addison and Ghoshray (2020) found that shocks to metal prices tend to be short-lived and cyclical for metals such as lead, manganese, nickel, steel, tin, zinc, and silver. However, for copper, gold, and platinum, price shocks are unlikely to dissipate quickly and could be long-lived or permanent.

Consumer behavior and the legislative environment will both shape the future demand for PGMs. The demand for vehicles may permanently change as a result of the Covid-19 pandemic. It is possible that even after the pandemic is over, more people will work from home and overall commuting will decline as the world has gotten used to a “new norm.” For example, during Covid-19, traffic declined by around 80% in many European cities, which meant that a typical car was only in use 1% of the time (Fengler 2020). This could lead to a long-term decline in demand for automobiles. If it is indeed a secular market, the implications of these changes would have a significant effect on the nearly 200,000 workers employed by the PGM sector and would face increasing job insecurity. Inversely, an increase in demand clean vehicles could prevent a long-term secular decline. Fuel cells generate electric power—and they are similar to a battery but do not need recharging and can run indefinitely when supplied with fuel. The cells produce electricity by combining hydrogen (the fuel) and oxygen (from the air) over a catalyst such as platinum or palladium.

### Impact of technological changes on PGM demand

The platinum group metals (PGMs) are comprised of coupled elements that, due to chemical similarities, always found together in deposits (Wellmer 2008). PGMs include light metals (ruthenium, rhodium, and palladium) and heavy metals (iridium, osmium, and platinum), and their primary function is as catalysts in the chemical and automotive industries, for the cleaning of exhaust emissions. They are chemically similar and, thus, are co-precipitated in the ore creation process. As a result, they are found, mined, and processed together (Renner and Wellmer 2020). The share of each metal in a deposit can vary. Each element is produced in different quantities, depending on the characteristics of the ore deposits. These ratios are rarely aligned with market needs (Kesler 1994). Within families of coupled metals, there are only one or a few elements that are key drivers for the production level (Renner and Wellmer 2020).

### Literature review

According to Surujrlal et al. (2014), between 2000 and 2012, the platinum group metal basket price per platinum ounce sold increased at a cumulative average growth rate (CAGR) of 8% in South Africa in rand terms. During the same time frame, industry cash operating costs per platinum ounce increased at a compound annual growth rate of 15–18%. The increased operating costs were largely a result of higher-than-inflation input costs including wages, electrical components, electricity, explosives, support material, reagents, and diesel. When coupled with anticipated uncertainty of the operating and market environments in the medium to long-term, the changes required firms to re-evaluate their operating strategies in an oversupplied market. As part of this process, choices and tradeoffs must be undertaken that shift firms to a different operating trajectory while providing stability and sharpening competitive advantage in a largely undifferentiated industry.

Part of reshaping firms’ strategies requires addressing both internal and external risks. Internal risk can be addressed by making organizational changes, careful implementation, and strong monitoring. External risk, driven by the uncertainty in the environment, is partly mitigated by anticipating potential challenges in the operating environment. A range of tools, techniques, and approaches can be deployed as part of the strategic planning process in the mining industry.

Commencing in 2010, Anglo Platinum, Impala Platinum, and Royal Bafokeng Platinum began struggling with profitability, while Lonmin was unable to recover from the Marikana Massacre and was acquired by Sibanye-Stillwater during the course of this research. According to Fennell and Alexander (1987), there are two “sets” of instruments that firms have used to reduce risk—buffers and bridges. Firms use buffers when they seek to protect core business activities from supply-side and demand-side volatilities, by shifting their overall business strategy. Firms use bridges to manage their environment through various negotiation tools, which can include instruments to improve community relations, such as expanding corporate social responsibility spending, paying royalties, and engaging in local procurement to build linkages with local communities. This paper assesses how PGM mining firms have used buffers and bridges to reduce their risk exposure as the industry undergoes a transformation.

### Literature gap

Little research has been done on how mining firms are responding to fiscal sustainability challenges, while also facing pressure to more equitably distribute mining-generated benefits to communities surrounding the mines, particularly within the South African context. Although work has been done on various bridging mechanisms such as royalties (Manson and Mbenga 2003) and corporate social responsibility (Rajak 2011, 2012) within the PGM sector, a gap remains with how firms balance protecting their core business activities and profitability, with meeting external expectations for supporting communities. The mining sector is inherently volatile and vulnerable to exogenous factors, such as supply and demand shocks. Understanding how mining firms bridge and buffer amidst commodity cycles offers valuable insight into how the sector can remain sustainable, both financially and in terms of maintaining their social license to operate. In Ernst and Young’s 2019–2020 survey of mining firms globally, a social license to operate was ranked the single largest risk facing the mining sector for the second year in a row. In 2018, Paul Mitchell, the firm’s mining and metals advisory leader noted,

License to operate has evolved beyond the narrow focus of societal and environmental issues. There are now increasing expectations of shared value outcomes from mining projects. Any misstep can impact the ability to access capital or even result in a complete loss of license – particularly in light of the increased use of social media, which makes potentially negative publicity more globally visible than ever.

Thus, this article aims to understand how PGM firms are utilizing Fennell and Alexander (1987) instruments—buffers and bridges—to protect their core business activities in what appears to be an increasingly secular market for platinum while also maintaining their social license to operate at a time when there are increasing pressure for firms to generated shared-value outcomes. The main premise of shared-value outcomes is that firm profitability and the health of communities are mutually dependent. This article constructs a novel case study and analyzes how firms have responded to endogenous and exogenous shocks by shifting their strategies to both protect their core business activities while also meeting the expectations of stakeholders to equitably distribute mining-generated benefits. The article constructs a case study using five sets of data to assess (i) what supply and demand shocks firms are facing; (ii) how they are reshaping their strategies and undertaking activities to protect the firm from these shocks; (iii) what challenges firms face with reaching shared-value outcomes; and (iv) how firms are undertaking activities to improve shared-value outcomes. This is a novel approach to constructing a case study within the extractive sector and offers valuable insight into firm behavior, as they try to balance profitability with improving shared-value outcomes.

## Methodology

Behavioral theory of the firm fits within the field of organizational economics—a multi-disciplinary field that draws from the larger field of economics, while also considering organizational behavior, strategic management, law, and other areas. Mahoney (2004:50) argues that, “If organizational economics and strategic management are to deal with uncertainty, they will have to understand how humans in fact behave in the face of uncertainty and by what limits of information and computability humans are bound.” To understand why firms do what they do, it is important to understand both the rational factors (such as high production costs) as well as the psychology behind it (such as why their strategies of community engagement have been largely ineffective, despite royalty payments). Organizational economics and strategic management are shaped by history of the risks, challenges, and consequences that firms have faced over time. In the case of South Africa, this means that firms make decisions based on the history of industrial action and community conflict and their impact on profitability, experiences trying to mechanize existing operations, and experience with supply and demand shocks.

Simon (1947) proposes a theory of human choice and decision-making that aims to bring together both rational aspects of choice that have historically been the primary area of concern for economists and the properties and limitations of the human decision-making mechanisms that have attracted the attention of psychologists. For example, the effect of Apartheid on mining communities must be factored into understanding community-firm challenges. Building on Simon’s approach, this article uses a variety of data sources to construct a case study on how PGM firms have undertaken decision-making amidst a range of supply and demand shocks. It uses a mixed methodology approach to understand how the rational side of decision-making and limitations of human decision-making have rendered some buffering and bridging activities more successful than others. It uses multiple data sets for both complementarity (seeking elaboration from results from a single method) and expansion (to extend the breadth and range of inquiry beyond a single method) (Greene et al. 1989).

### Data sets for buffering

The first data set was market intelligence data, acquired from the Metals and Mining service on the Standard and Poor’s (S&P) Global Market Intelligence platform. S&P Global Market Intelligence integrates financial and industry data, research, and news into tools that can help track performance, identify investment ideas, understand competitive and industry dynamics, perform valuation, and assess risk. The global metals and mining solution offers a comprehensive source of global exploration budget details, development, production, mine cost analysis, acquisitions activity, commodity market forecasts, credit risk assessments, and climate risk evaluation.^2^ For this article, the platform provided valuable data on production cost breakdowns for many PGM mines as well as firm-level annual revenue data which was important to calculate the cost of strikes, which was important to quantify the cost of industrial action. All S&P data was acquired through a short-term paid membership.

### Data sets for bridging

The second data set was trade data from the United Nations Comtrade Database, which is a repository of official international trade statistics and relevant analytical tables. Data was used for HS codes 843069 (machinery; moving/grading/levelling/scraping/excavating/extracting machinery, for earth/mins./ores (excl. of 8430.10–8430.49), other than self-propelled) and 843050 (machinery; for handling earth, minerals or ores, self-propelled, n.e.c. in heading no. 8430). This data was valuable to understand the extent of local manufacturing of mining machinery versus utilization of imported machinery. This was a proxy to understand the extent to which downstream linkages have been formed.

### Data sets for buffering and bridging

The third data set was acquired through interviews undertaken at three underground and open-pit mines with mine workers and mine-level management as well as with c-suite, human resource development, and sustainability executives at headquarters offices of four of the largest PGM mining firms. A total of 71 semi-structured interviews were conducted between March 2018 and February 2019 as part of the larger thesis research—of those, data was drawn from 31 of them for this article. A snowball sampling approach was taken, which is a non-probability sampling technique in which existing contacts recruit future subjects through their social networks. This sampling technique is often used where future subjects are difficult for researchers to access, both due to seniority and/or sensitive topics. In this case, initial subjects were established during previous research, in which the author lived in Rustenburg, the PGM mining hub of the region, for a 1-year period in 2014. Interviews for this study were conducted in 2018 and 2019. The snowballing sample approach allowed the researcher to build a stronger level of trust, because she was recommended by someone that they trust. Some topics such as retrenchments are more sensitive, and people often prefer anonymity.

The fourth data set was gathered from the Neil Coleman Collection at the South African Historical Archives. The Neil Coleman collection was commissioned by the Labor Research Committee between 1979 and 1981. A team headed by Neil Coleman conducted research on influx control, migrant labor, and conditions of the labor force in various industries for the period between 1930 and 1980, inclusive. There were 39 boxes of data within the collection and this information was useful in terms of contextualizing challenges facing firms over time.

The fifth data set was both internal and external reports and presentations shared by firms. A total of 16 reports and documents were analyzed, which included financial and sustainability reporting shared by 4 firms, industry performance reports shared by Minerals Council SA, and internal strategy documents shared by two large PGM producers on labor and mechanization.

## Findings

### Supply and demand risks that require buffering

Since platinum prices began declining in 2008, firms in the PGM sector have adopted buffers, which are designed to protect core business activities, by developing strategies to increase their overall efficiency by reducing labor costs (thereby also reducing exposure to labor strikes) and increasing productivity. This section focuses on two supply-side risks, high labor volatility and high labor costs, and one demand-side risk, the shift from platinum to palladium, that firms have adopted buffering strategies against.

#### Supply-side risk: high labor volatility

Strikes have been a central part of the mining narrative in South Africa. In 1946, mine workers went on strike for a wage increase. Within a week, 9 were killed and 1248 were wounded as the police and army viciously attacked strikers. In 1987, 340,000 mine workers went on strike with the National Union of Mineworkers (NUM), who argued for a 40 to 55% wage increase, concessions concerning holiday leave, and danger pay and death benefits. The strike became very violent. During 3 weeks of strikes, 9 were killed, 500 injured, and 400 arrested. The strike did not end successfully for workers—Anglo American threatened to fire its entire workforce and workers went back to work defeated (Davenport 2013). In 2012, the South African Platinum sector made international headlines during the Marikana Mining Massacre. The strikes began at Impala, where rock drill operators (RDOs), one of the lowest skill jobs in a conventional mine, demanded a wage increase and improved housing. As the strikes progressed and spread, 44 people were killed at Lonmin (11 in the first instance, 33 in the second), making it the largest use of state police force since the 1960 Sharpeville Massacre.

Still, workers felt their concerns were unaddressed. By the end of 2013, firms understood that a dissatisfied workforce was a threat to their profitability. However, 2014 saw a strike of different magnitude—it lasted so long that it threatened both firm financial sustainability and South Africa’s macroeconomic stability. Previous strikes had not translated to macroeconomic impact. But 2014 did—platinum is the single largest export within the mining sector, and the halt to production adversely affected the country’s economic stability.

It started in January 2014 when nearly 70,000 PGM mine workers went on strike. The majority of them came from the three largest mining houses—Anglo Platinum, Lonmin, and Impala—all based in the Rustenburg area of the North West Province. These workers belonged to a newly formed labor union, the Association of Mines and Construction Union (AMCU), and the union demanded a salary increase from R5000 to R12500 per month for their workers. However, PGM companies called this request unfeasible and offered a maximum 10% wage increase. After 5 months of striking, the PGM companies and AMCU settled for a pay increase distributed over 3 years. The agreement stated that workers earning less than R12500 would get a R1000 increase in 2014 and 2015 and an R950 increase in 2016. The new minimum salary would be R8000 per month. However, by the time the deal had been struck, it became the longest and most expensive strike in South African history. The strikes lasted 152 days. During this time, the mines lost 48.5% of platinum production, taking 440,000 oz out of production. The three largest producers suffered an annual revenue loss of R24.1 billion during the strike and workers took a further loss of R10.6 billion in wages. The mining strike stifled economic growth—GDP only grew by 0.6% during the first quarter of 2014, and GDP declined by 1.3% for 2014.

**Fig. 1:**
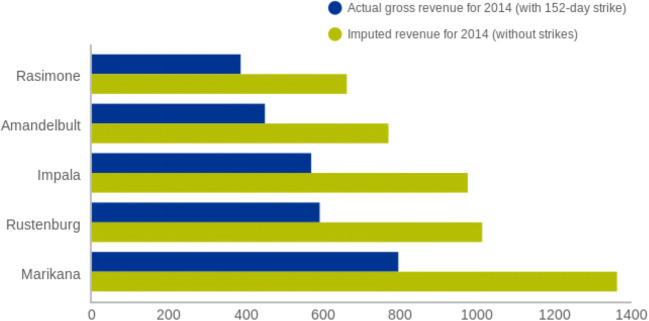
Impact of 2014 industrial action on mine revenue. Source: author’s calculations using S&P data

Figure 1 shows the impact of the 152-day strike on five large mines—Marikana, Rustenburg, Impala, Amandelbult, and Rasimone. The losses ranged from 48 to 64% for the year. After the strike, Anglo Platinum announced its plan to sell four mines and two joint ventures. Within 5 years of the strike, Anglo Platinum, Lonmin, and Impala had exited most of these operations—Sibanye Stillwater acquired the Marikana Mike through an acquisition with Lonmin and also purchased the Rustenburg Mine from Anglo Platinum. Impala announced the closure of five of its Rustenburg operations. It was not an end to mining, but rather the tipping point for many firms to move from labor-intensive to capital-intensive mines, for financial sustainability.

**Fig. 2:**
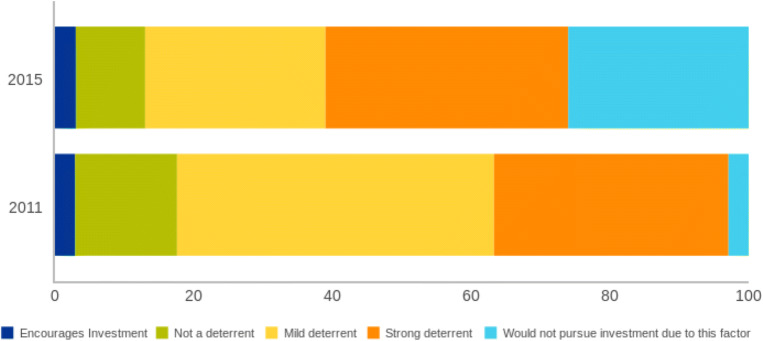
Perceptions of labor regulations/employment agreements and labor militancy/work disruptions on investment. Source: Fraser Institute

Every year, the Fraser Institute, a Canadian think tank, runs a survey of mining and exploration companies to assess how mineral endowments and public policy factors affect exploration and investment. In 2011, before the Marikana Massacre (2012) and 152-day strike (2014), just 3% of mining and exploration firms surveyed reported that they would not pursue investment in South Africa due to labor regulations, employment agreements, and labor militancy/work disruptions. Most viewed it as a minor issue—45% reported that it was a mild deterrent to investing in South Africa. By 2015, over 60% of firms cited that labor challenges were a “strong deterrent to investment” or a reason that they would “not pursue investment” (Fig. 2).

#### Supply-side risk: high labor costs

**Fig. 3:**
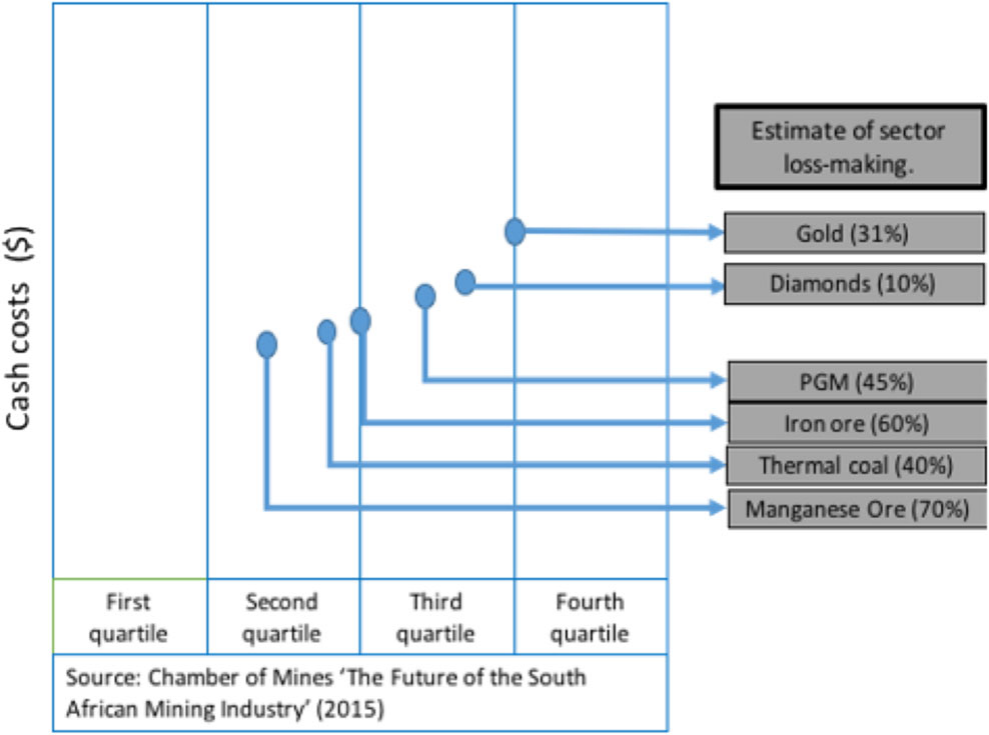
Production costs of various minerals and metals in South Africa. Source: Chamber of Mines (2015)

An analysis of the S&P Market Intelligence data shows that South African PGM mines have amongst the highest production costs globally. Of the 12 PGM mines in the highest two quintiles for production costs, 10 are located in South Africa. Of the 4 mines that are in the lowest quintile for production costs, just 1 is in South Africa (Table 1). By 2015, 45% of all PGM mines in South Africa were estimated to be making a loss by the Chamber of Mines (Fig. 3).

**Table 1:** Cost competitiveness of PGM mines

Production costs	Number of South African PGM Mines	Number of PGM mines in the world
First quartile	1	4
Second quartile	4	8
Third quartile	5	7
Fourth quartile	5	5

Source: S&P Market Intelligence

**Fig. 4:**
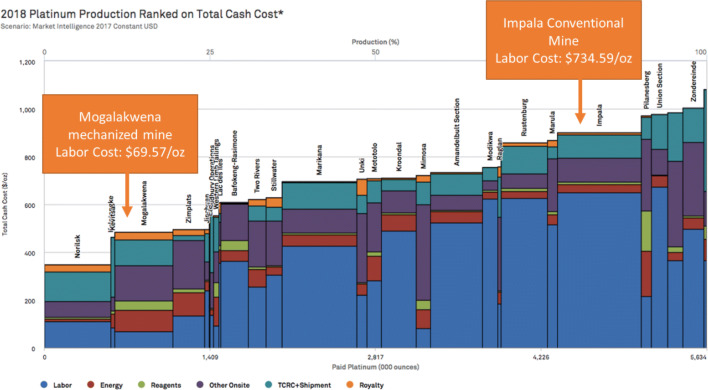
Production costs of PGM Mines. Source: S&P Market Intelligence

In light of declining platinum prices and unprofitable operations at a number of mines, further analysis of production costs reveals that labor costs play a significant role in reducing overall cost competitiveness. At Impala Platinum’s conventional PGM mines, labor costs accounted for the biggest production cost, amounting to $734.59 per ounce in 2018, with total production costs at $900 per ounce. Comparatively, at Anglo Platinum’s flagship Mogalakwena operation, which is highly mechanized and has some of the lowest operating costs in the industry, labor costs are just $69.57 per ounce, with total production costs at $500 per ounce (Fig. 4). Over the last decade, platinum prices have often been below $900 per ounce, indicating that many conventional mines were grappling with long-term unprofitability.

There is also a substantial productivity difference between mining methods. Amandelbult is a conventional mining operation owned by Anglo Platinum. In 2019, the operation employed 15,663 people (9.1% of whom were contractors) many of whom are low or semi-skilled and lived proximate to the mine. An average of 57 oz of PGMs was produced per person employed. At Mogalakwena, the mechanized operation, 2208 people (12.3% of whom were contractors) were employed in 2019. An average of 550 oz of PGMs was produced per employee. Mogalakwena has concentrated its efforts on increasing the efficiency of its capital investments, including drills, explosives, diesel, shovels, and mining trucks. Since 2012, drill penetration has improved by 35% while mining truck utilization has increased by 15%.

#### Demand-side risk: shift in global demand

**Fig. 5:**
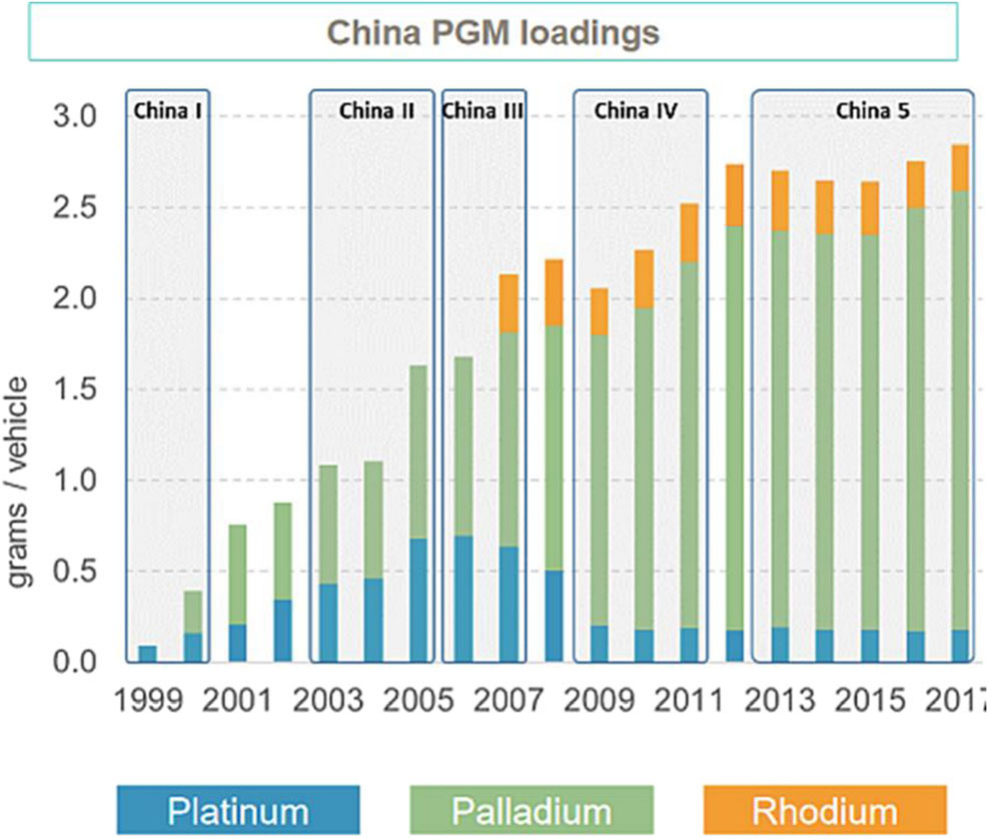
Impact of Chinese emissions standards on PGM demand. Source: World Platinum Investment Council

**Fig. 6:**
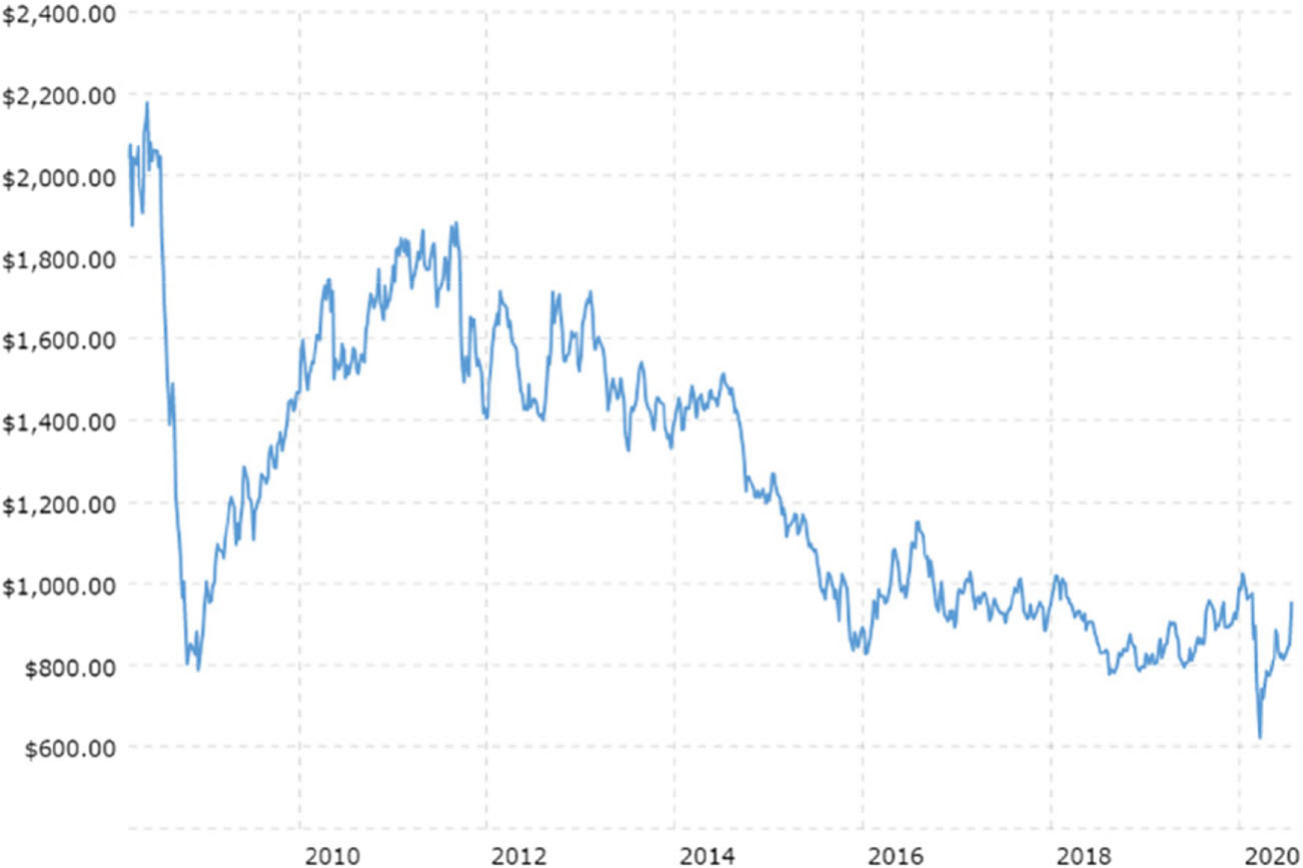
Platinum prices between 2008 and 2020. Source: Macro Trends

**Fig. 7:**
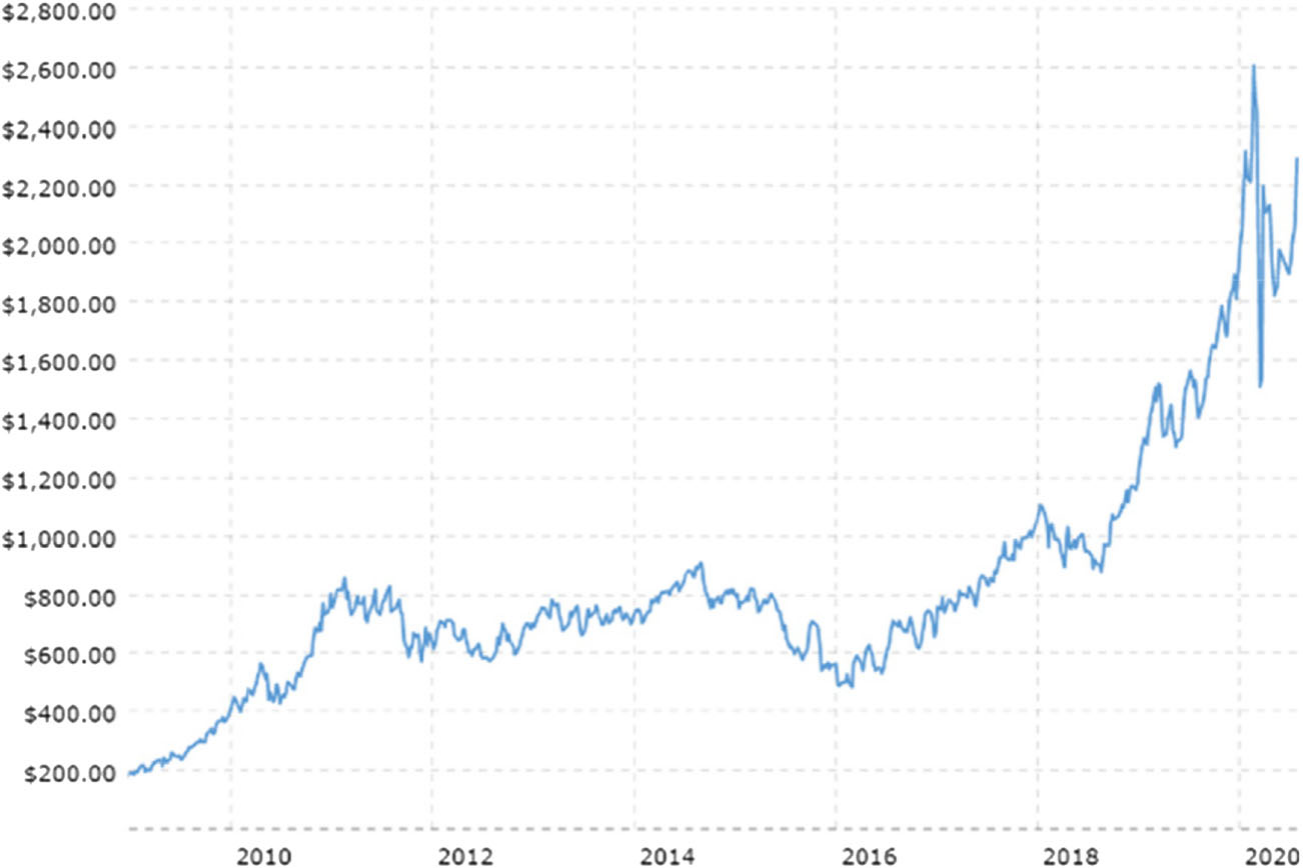
Palladium prices between 2008 and 2020. Source: Macro Trends

Within the PGM group, the driver has historically been platinum. However, over the last decade, the platinum market has been oversupplied for two reasons: first, there has been an influx of recycled platinum from East Asia, and second, the push for tighter emissions legislation has favored palladium and rhodium over platinum. Platinum is largely used for catalytic converters in diesel cars, while palladium is used in gasoline engines. Since the scandal in 2014, in which software in diesel cars from a number of manufacturers was manipulated to enable vehicles to pass air pollution tests despite producing substantially more pollution than allowed (sometimes more than ten times as much nitrogen oxide than was permitted by European emission limits), there has been a significant decline in demand for diesel vehicles.^3^ Figure 5 shows how Chinese emissions standards have had a significant impact on PGM Demand, as palladium loads have exponentially increased while platinum loadings have decreased substantially. Thus, prices for palladium have over tripled between 2016 (average $614/oz) and 2020 (average $2074 per ounce) and prices for rhodium increased from $1000 per ounce in 2015 to $14,800 per ounce in 2020 while prices for platinum have declined from a high of $2167 per ounce in 2008 down to $1384 per ounce in 2014 and a further fall to an average of $856 per ounce in 2020^4^ (Figs. 6 and 7). Although rhodium is substantially more valuable than palladium, firms in South Africa have focused on palladium given that rhodium typically accounts for less than 10% of the PGM ounces mined, while palladium and platinum together account for 89–94% mined ore. With the exception of Mogalakwena and Waterberg, the latter of which is a brand new operation under construction, palladium typically accounts for 25 to 35% of PGM deposits (Table 2). But a number of mining houses mine palladium at close to a 1 to 1 ratio with platinum, indicating that the palladium deposits are being extracted at a disproportionately higher rate compared to platinum.^5^

**Table 2:** Palladium deposits at various PGM mines

Mine	Share of palladium (%)
Bafokeng Rasimone	26.7
Tumela	29.3 (average of Merensky and UG2 reefs)
Union	29.0 (average of Merensky and UG2 reefs)
Mogalakwena (flagship)	49.3
Waterberg (new flagship under construction)	63.0

Source: Firm reporting

### Buffering strategies

After platinum prices began declining, mining companies began reassessing their strategies to identify areas to cut costs. Retrenchments became a routine part of the PGM operational discourse around 2010. The 152-day labor strike, which brought production to a halt in 2014, forced many companies to reassess their high reliance on labor. Interviews with executives at the four largest mining PGM companies revealed a reoccurring trend—firms are hesitant to reduce labor at existing operations by modernizing them or mechanizing them, in fear of retaliatory strikes that could stop production. Instead, firms are constructing and expanding operations that were mechanized from the beginning and closing or selling conventional labor-intensive assets. As one chief executive officer stated, “If we start with a mechanized mine, it does not upset people. Communities and workers do not revolt if we build a fully mechanized mine – they revolt if we retrench workers from an existing operation and replace them with technology.”

This strategy has been evident in the operational decisions made by PGM companies. Following the 2014 strike, Anglo Platinum announced plans to sell its most unprofitable conventional PGM assets in South Africa, including its Rustenburg Mine, Union Mine, and two other joint ventures, so it could focus on less labor-intensive operations. Most notably, Anglo Platinum has focused on the expansion of its highly mechanized Mogalakwena operation, often called the “crowned jewel” of the firm’s portfolio for its high production and low operating costs. Other large mining houses have undertaken similar moves. In 2017, Royal Bafokeng Platinum announced that there would be a retrenchment of over 500 workers as the firm restructured its unprofitable Bafokeng Rasimone Platinum Mine (McKay 2017). During this same time, it was expanding its Styldrift Mine, a fully mechanized underground operation, to produce 230,000 tons per month with a life of mine over 30 years. In 2018, Impala Platinum, the world’s second-largest platinum producer, announced the closure of five unprofitable conventional operations in the Rustenburg area and announced the retrenchment of 13,000 workers, amounting to one-third of its total workforce. However, the company has been simultaneously investing in the construction of the Waterberg Project, a fully mechanized mine in Limpopo that will use 400 trackless machines to carry out operations.

The share of rhodium and palladium in various locations has also been a key factor in selecting new mine operations. Historically, the platinum group metals family has largely been driven by its namesake, platinum. However, given the shift in global demand resulting from the automotive industry, more firms are opting to pursue operations in areas with high palladium concentrations. Table 2 shows that the flagship operations for the two biggest PGM producers in the world—Mogalakwena for Anglo Platinum and Waterberg for Impala—are much more palladium rich than older operations. Likewise, an article published by Mining Weekly in 2018 entitled “PGM producer focuses on Palladium in Limpopo” states that “Platinum and palladium mining company Platinum Group Metals is refocusing its business on the large-scale, bulk mineable Waterberg project, in Limpopo, South Africa, which is dominated by palladium and has reserves in platinum, rhodium, gold, copper and nickel.” (Breytenbach 2018). Platinum was positioned as a byproduct of the mainstay of palladium.

### Risks that require bridging

**Fig. 8:**
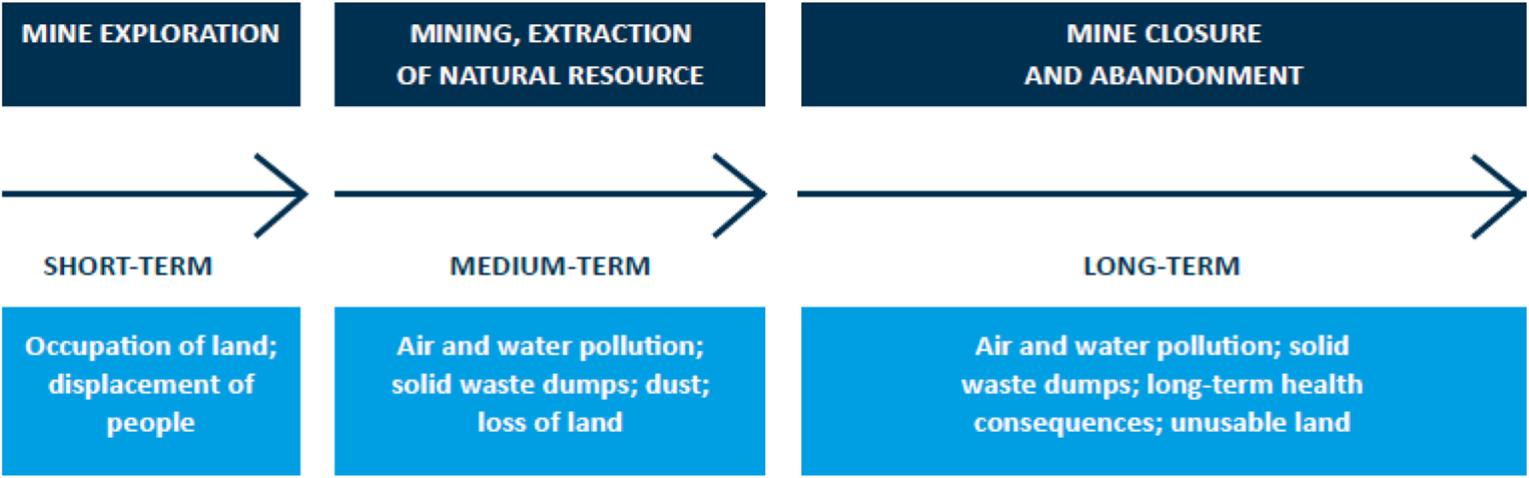
Impact of PGM mining on communities across the life of a mine. Source: World Bank (2019)

A central challenge of the extractive sector is that benefits accrue predominantly at the national level while disruptions are invariably highly localized close to the resource (Macdonald 2018:1), including land encroachment, environmental consequences, and adverse health effects across the life of a mine and beyond (Fig. 8). The inequitable distribution of risks, impact, and benefits of mining is a driver of community-company conflict. Communities have not perceived that the benefits that they have received from mining activities have sufficiently compensated them for the consequences. However, in recent years, mining companies have intensified efforts to correct this imbalance, partially due to pressure from governments, shareholders, and communities.

To ameliorate this imbalance, financial institutions and investors have focused on strengthening environmental, social, and governance (ESG standards). Once perceived as a “nice to have,” ESG and sustainability expectations have become a “must have” in recent years. Although most sectors are scrutinized for ESG, the mining sector is on a deeper level given the negative impact of mining on both people and the planet. A variety of mechanisms have been used to achieve this, including corporate social responsibility (CSR) and the social license to operate, both of which have gained great currency in recent years and are used as instruments to improve the standard of living of local communities (Macdonald 2018). Firms engage in bridging activities to adapt their organizational behavior to conform with external expectations and have shared-value outcomes from mining projects.

#### Land encroachment

Land encroachment is an inherent structural challenge of mining globally given that mining firms are extracting from land that that is home to communities. In South Africa, there has been an intense struggle over communal land. Chiefly authorities often disproportionately benefit from mining licenses, as they often become major shareholders or get royalty payments with little accountability. For example, in the late 1960s, Amplats Union had come to an agreement to pay the Bakgatla tribal chief to extract from the Spitskop Mine. However, local residents were angry, claiming that they had not been consulted about the agreement or compensated for the loss of their grazing and farming lands. This mine became known as the “Sinkgalaleng” mine, which translates to “don’t undermine us.” By the late 1990s, the village’s youth protested that the village had never benefited from infrastructure development or employment from the mine. Tensions remerged in 2006 when rumors circulated that Chief Nyalala was in negotiations for a direct equity stake in Union, via a conversion of the tribe’s royalty stream. Infuriated, villagers argued that they did not know that Spitskop Mine had been paying royalties. To exacerbate things, it then emerged that the Union had been illegally encroaching on land outside of the parameters of the lease area (Capps and Mnwana 2015).

Likewise, in 2007, residents of Ga Puka and Ga Sekhaolelo filed a lawsuit in the High Court Transvaal Provincial Division against Potgietersrust Platinum’s Ltd., a wholly owned subsidiary of Anglo Platinum and nine other defendants (including the South African Government). The plaintiffs requested an injunction prohibiting Anglo Platinum from interfering with the residents’ land rights in an area where they were expanding their mining activities. The plaintiffs sought to set aside the relocation agreement Anglo Platinum made by arguing that the institution that was supposed to represent residents was under the influence of Anglo Platinum. The plaintiffs also argued that Anglo Platinum was forcibly relocating residents because individual householders signed relocation agreements under duress. Though the case was dismissed by the court, it is indicative of the tension arising from land encroachment.

The land encroachment struggle has intensified with mechanized mining, which has drastically increased the rate of extraction. At Mogalakwena, open-pit mechanized mining takes out 85,000 tons in one shift, and there are four shifts each day. Conventional mining takes out 85,000 tons each month. As a result, the expansion into neighboring communities has been expedited.

The loss of land can translate to loss of livelihoods for those who are dependent on it, such as those in the agriculture sector. This loss has been quantified in South Africa’s coal sector. A study by the Bureau for Food and Agricultural Policy (2012) found that at the current rate of coal mining, about 12% of South Africa’s high potential arable land will be permanently damaged by coal mining with a further 14% being subjected to coal prospecting applications. The study found that current coal mining activities could lead to a loss of 284,844 tons of maize a year, with an additional vulnerability coming from 162,736 tons grown in areas that are currently being prospected. This has marked effects on food security—the price of maize in the South African market could increase by 14% due to shrinkage in supply. In fact, if coal mining continues to grow, thereby extending the loss of maize-growing land, 240,000 ha of land could be lost, which would translate to a loss of 1.2-million tons of maize—enough to make South Africa a permanent maize importer (Baskaran 2019).

#### Environmental consequences

In addition to a loss of land, which can adversely affect livelihood by taking away land for agriculture, land encroachment also brings dangerous pollution close to home for communities around mines. Environmental pollutions stemming from solid waste from tailings and waste rock are felt by mining communities, both in terms of damage to land and water, as well as resulting health consequences. Water damage can come from acid mine drainage (AMD), which causes trace metals to leach out from the waste and into the water. Acid is carried from the mine through surface drainage or rainwater and deposited into local streams, lake rivers, and groundwater, severely damaging water quality. AMD can continue for hundreds of years. The second form of pollution is processing chemical pollution, which occurs when chemical agents—such as cyanide or sulfuric acid—is used to separate the mineral from the ore—spills, leaks, or leaches from the mine site to nearby water bodies. Such chemicals can be highly toxic to both humans and wildlife (World Bank 2019). Approximately 1.6 million people live in informal and formal settlements on, or near, mine dumps in South Africa. People in these settings tend to be historically disadvantaged and poor. Pollution from mine can adversely affect the health of nearby residents. PGM mines tend to have high rates of carbon dioxide, sulfur dioxide, and dust emissions. There are many health consequences of sulfur dioxide emissions, including decreased lung function, respiratory illness, alterations in pulmonary defenses, aggravations of existing, and susceptibility to new cardiovascular disease or chronic lung disease, and daily mortality. There is no safe level of exposure to sulfur dioxide. Consequences of dust exposure include respiratory problems, damage to lung tissue, cancer, and premature death (Cairncross 2014).

#### Health consequences

Beyond the adverse health effects resulting from environmental pollution, there are also other health consequences. Mining communities in South Africa have been epicenters for diseases such as tuberculosis (TB) and human immunodeficiency virus (HIV). In South Africa, the prevalence of TB among miners and ex-miners is estimated at 2500–3000 per 100,000 people (compared with 834/100,000 people in the general population), which is more than 10 times the World Health Organization’s threshold for a health emergency (World Bank Group 2018). A 2014 study by the World Bank found that 82% of mining companies gave employees access to a mine clinic or a hospital. However, facilities were lacking in terms of diagnostics—just 67% utilized a cough questionnaire (a basic test to identify a nagging cough, which is a key symptom of both silicosis and active TB); and not all companies had access to chest x-ray machines and sputum tests. It is unsurprising that nearly 70% of TB cases in the mines are missed or go untreated. Additionally, while many companies have policies that cover diagnostic and treatment for workers, there are still two major gaps in coverage—contractors and family members. The World Bank (2014) study found that contractors did not have the same access as regular employees to health services. Specifically, 33% of companies had no written policy in terms of providing TB services for contractors and those without written policies did not necessarily allow contractors to utilize their TB services. Second, only regular employees are generally given access to the free care provided by mining companies’ health facilities—their family members are required to purchase separate insurance to be treated there. Given the airborne nature of TB, it is common for it to spread to family members but treatment for them could be prohibitively expensive (World Bank 2014).

### Bridging strategies

Given the many adverse effects that mining communities have faced—losing land to mining operations, facing environmental harm, pressure from labor strikes, and mining-related illnesses—without being fully compensated for them, there has been increasing pressure for a transformation of the sector over the last two decades. In 2002, the Minerals and Petroleum Resources Development Bill (MPRDA) was gazette in South Africa. One of the objectives of the Act is to “Ensure that holders of mining and production rights contribute towards the socioeconomic development of the areas in which they are operating.” The same year, the Mining Charter was drafted in South Africa. One of the core objectives of the Mining Charter was to, “Promote employment and advance the social and economic welfare of mining communities and the major labor sending areas.” Thus, for firms to negotiate their place in post-Apartheid South Africa where the transformation of the sector was a priority, they had to increase their support for communities surrounding the mines. A favorable perception of the firm is critical to have social buy-in from local communities. Firms began to increase their bridging efforts. Bridging “occurs as firms seek to adapt organizational activities so that they conform with external expectations.” (Meznar and Nigh 1995: 976). Firms use bridges to manage their environment through various negotiation tools, which can include instruments to improve community relations, such expanding corporate social responsibility spending, paying royalties, engaging in local procurement, employing locals, and building infrastructures such as schools, hospitals, and roads.

Without sufficient bridging, mine operations can be halted by protests. One example is Twickenham Mine, an Anglo Platinum mine located in the Sekhukhune District Municipality in Limpopo Province, with the operations area covering Tubatse, Fetakgomo, and Makhuduthamaga local municipalities. These are some of the most impoverished communities in South Africa, with less than 25% of eligible adults in full employment. Twickenham Mine began development in 2001. By 2014, as platinum prices declined, Anglo Platinum opted to pause its expansion projects for Twickenham and, in 2014, chose to suspend operations, placing it on “care and maintenance.” Under this operational level, mine production declined to less than 5%—just enough to keep it from going into closure. Anglo Platinum retrenched nearly all of its 2000 workers, retaining a skeleton staff of 121 people and turned the shaft into a research and development (R&D) laboratory for new mechanized technology. The retrenchment of workers was not taken well by the surrounding community, who vehemently protested the decision. Tires were burned on the single road that provides access to the mine, while protesters tampered with the electricity supply, without regard for anyone who may have been underground. Surrounding communities threatened an armed takeover unless Twickenham Mine employed a minimum of 5000 locals, which was a far cry from the 121 staff and 500 R&D contractors who worked on site. A field visit to Twickenham Mine was undertaken for this research, and fear was a constant theme amongst interviewees—fear that they would be underground when electricity cables were cut by the community, fear of an armed takeover of the mine, and fear that they would not be able to exit the mine if the single road into the mine was blocked off by protestors.

#### Royalties

Royalties are paid to communities who own the land from which minerals are extracted. However, royalty negotiations can be difficult. One of the major wins for communities around PGM mines came in 1999 after a nearly 5-year court battle between the Royal Bafokeng Nation and Impala Platinum. Impala settled and the Bafokeng won 22% of pre-tax profit as their royalty share. These revenues have been used to finance Royal Bafokeng Sports (RBS), which is responsible for sports development, the Royal Bafokeng Administration (RBA) which works to ensure that infrastructure and services are aligned with the long-term vision, and Royal Bafokeng Institute (RBI), which works to improve education and learning amongst the Bafokeng people.

However, this has not necessarily translated to a satisfied community. Flomenhoft (2019) undertook a survey of people within the Bafokeng community to understand how satisfied they were with the services provided to them by the RBA, financed by mining royalties. The study found that 77.1% of people surveyed do not feel they are sufficiently benefitting. Unemployment, poor-quality services, and mismanagement of funds were the most common concerns. Less than 3% of respondents named problems with mines as the problem. This demonstrates that most survey respondents felt the traditional authorities have not used the royalties to sufficiently improve their quality of life.

This is a larger challenge across PGM mining communities. A report by Corruption Watch in 2018 found that there is a deep distrust in mining communities about royalty distribution. Two Anglo Platinum projects—GaPhasha and Booysendal—had such tension that community members “were frightened to go on record to speak about what they consider collusion between traditional authorities and mining companies.” The report goes on to say that billions of rand in mining royalties intended to improve socioeconomic development in mining communities are maladministered, squandered, or stolen. There is a lack of transparency around the negotiation and distribution of royalties with various stakeholders—traditional leaders, government, community members—trying to increase their share of royalties. This leads to mining communities continuing to live in severe poverty despite royalty payments.

#### Corporate social responsibility

The use of CSR is controversial. Rajak (2011:27) argues that it is part of the “ritualized performance of global corporate citizenship, that companies stake their claim to the moral capacity that such a status affords.” It projects firms as more than moral agents, but also as “vehicles” of sustainable development through business and empowerment. On the other hand, Mutti et al. (2012:221) note that it is “patronizing and paternalistic, when companies undermine knowledge and skills of local communities to identify their own needs and priorities. This leads to a lack of adaptation to local conditions, as companies appear to enforce their own vision of community ‘good.’” Although South African PGM firms have actively engaged with CSR, they have largely approached it as “ad hoc charitable donations to good causes” (Hamann 2004). For example, in its 2019 Sustainable Development report, Impala Platinum highlighted two core CSR projects—the electrification of 130 homes in Ga-Kgwete Village and 77 households in Ga-Mashishi and the firm’s sponsorship of swimming pool containers to curb the risk of drowning among children in disadvantaged communities.

Field interviews showed that when CSR projects conclude, an adverse effect can occur. For example, one mining executive interviewed had been removed from his post for a period of 12 months and given the task of setting up a CSR project to improve the schools around the mines. He found that teachers were often absent and/or disinterested in their teaching and learners were often disengaged from the learning process and very far behind academically. The firm financed and ran a CSR program that placed top math, science, and engineering university students in secondary classrooms to teach, learning outcomes improved. However, after the program stopped being run, scores fell again. Recent matric pass rates were 3.3% in pure math. The firm reported that the Department of Education (DBE) asked the firm to give them the money and the Department would implement the program. However, the firm declined given that the DBE has not made meaningful progress in schools in these communities. The executive noted that the education system was a “disaster” in communities around the mines, and although CSR projects could put a “dent” into challenging socioeconomic challenges, they were not sustainable solutions to structural problems.

#### Local procurement

Local procurement is the purchase of goods and services from local businesses. By integrating local communities into the supply chain, communities can be supported through skills development and job creation. Local procurement is a tool increasingly used by mining companies for four reasons. First, it de-risks company operations because communities are less likely to stop operations with protests if they are part of the supply chain. Second, it enables firms to comply with government regulations on the local content requirements, making it easier for firms to get and keep their operating license. Third, it provides benefits for local communities by generating business opportunities for which the mining firm can be the off-taker (IFC 2011). And finally, it can improve mining companies’ supply chain efficiency by reducing logistics costs.

The 2018 Mining Charter has prioritized mining firms utilizing local procurement wherever possible—it is the highest weighted contributor to overall compliance accounting for 40 of the 100%. Interviews with mining firms revealed that local procurement is still largely done through the procurement of low-value services such as cooking meals and cleaning. Procurement of mining machinery and other inputs is more difficult and rarer, given the limited manufacturing capacity and skills of local communities. In fact, both upstream linkages, which relate to the procurement of goods and services that mines need to produce, and downstream linkages, which result from further processing of the extracted commodity—have gone down in some areas.

In terms of downstream linkages, data from the United Nations Comtrade International Trade Statistics Database shows that when looking at mining machinery alone,^6^ trade balances have experienced a sharp shift. In 2006, mining machinery imports and exports were nearly equal—$11 million and $10.7 million, respectively. Within 3 years, the value of exported machinery declined from $10.7 to $9.6 million while the value of imports doubled from $11 to $22 million. By 2012, the value of imported mining machinery nearly doubled again to $41 million, while the value of exports experienced a more moderate increase to $22.2 million. Procurement of domestic services to run this machinery is also more difficult. Domestic skill constraints coupled with new technology have required an increase in foreign labor. In South Africa’s mechanized mines, imports include machinery from Sweden’s Sandvik and Epiroc, Croatia’s Doking, and Japan’s Komatsu. Given that local staff are not skilled at operating these technologies, foreign labor is being utilized in both mechanized underground (TM3) and mechanized open-pit (TM4) mining. For example, at Anglo Platinum’s Twickenham Mine, which is currently a research and development site for mechanized technology, a team of Croatian workers has been on site year-round to operate the imported remote-controlled blasting equipment.^7^

In terms of upstream linkages, catalytic convertors are the primary end product of PGMs. But local manufacturing of catalytic convertors has also declined over time. The catalytic converter industry in South Africa developed largely as a result of the Department of Trade and Industry’s MIDP incentive scheme, which was discontinued in 2013. The current projections for local PGM beneficiation via catalytic converters in South Africa reflect a downward trend, due to production being moved to other countries as a result of policy uncertainty around government incentive programs. The industry is capacitated to support 23.7 million units per annum. At full capacity, the industry would represent 19% of global auto catalyst production. However, South Africa currently manufactures just 8% of the world’s catalytic convertors despite holding 38% of the world’s palladium and 75% of the world’s platinum. Although the global market for catalytic converters is expected to grow at a CAGR of ~2.9% over the next 5 years, South African PGM producers have opted for long-term contractual agreements with major refineries and manufacturers in Europe (UK, Germany, Switzerland), Japan, China, and North America. China is the largest supplier of automotive catalytic converters, with a production market share nearly 28%, followed by Europe, which had a market share of nearly 26% in 2015.

## Discussion

In recent years, firms have worked to reduce their exposure to volatility—resulting from both endogenous and exogenous supply and demand shocks—by buffering and bridging. Firms have been very proactive about engaging in buffering to protect their business activities from exogenous factors such as fluctuating platinum prices. They have undertaken strategic long-term plans to de-risk their mining portfolio by selling off or closing conventional mining assets and focusing new mechanized operations on areas with high concentrations of palladium. Bridging efforts have been less successful at achieving shared-value outcomes—the findings show that royalties are often vulnerable to maladministration which prevents the benefits from reaching communities; CSR is inherently unsustainable and stopping projects can often trigger adverse rebound effects, as demonstrated by the education CSR project discussed earlier. Additionally, CSR projects are often ad hoc in nature—like creating swimming pool containers—which makes sustainability more difficult; and finally, local procurement is often limited to low-value-added services such as cooking and cleaning because of limited manufacturing and skills in surrounding communities. Downstream and upstream linkages have declined over time. Additionally, because of the high regulations in the Mining Charter in six areas—ownership, mine community development, employment equity, human resource development, inclusive procurement and supplier and enterprise development, and housing and living conditions—bridging efforts can often feel like firms ‘complying’ to meet requirements, rather than a genuine desire to distribute mining-generated benefits more equitably.

Bridging efforts are further hampered by the fact that mining communities often prioritize employment over other benefits such as service provision. As discussed earlier, at Twickenham Mine, armed protests stemmed from a request to employ a minimum of 5000 locals. Similarly, Flomenhoft (2019) found that employment was identified as the most important benefit of mining activities in a survey of a PGM mining community. However, in the mechanized era of mining, employment is declining. In October 2019, Minerals Council SA then announced that in the PGM sector, 90,000 of 168,000 jobs—or 54% of the sector’s employment—were now at risk. This makes it more important than ever for labor-intensive, non-mining activities such as manufacturing or agriculture to be developed in PGM mining communities.

Firms buffering activities have substantially improved their financial sustainability. After consecutive years of unprofitability and selling loss-making assets, the shift to mechanization and focus on increasing palladium production paid off. In February 2020, Anglo Platinum released full-year results for 2019. There was a 145% increase in headline earnings to R18.6 bn. The dividend payout was R14.2 bn, which amounted to 76% of headline earnings and in 1 year, was double the amount the firm paid for the previous 10 years. However, substantial conflict around some of Anglo Platinum’s operations (such as Twickenham) highlights the need for increased bridging efforts to reduce tensions and make a more equitable distribution of benefits. As the PGM sector continues its shift to mechanization and closes labor-intensive conventional operations, there is likely to be more conflict with surrounding communities.

## Conclusion

This article has assessed what risks PGM firms face and how firms pursue buffering and bridging activities to reduce their exposure to these risks. Compounding supply and demand shocks have become a central part of the PGM narrative over the last decade. Supply shocks have come from production-stopping strikes while demand shocks have come from a sharp decline in demand for platinum, resulting from an increase of recycled platinum from Asia and the diesel scandal in Europe. Given that this could mark the transition to a secular market, like tin in Cornwall, firms have focused on mining operations that have large deposits of palladium and that require large capital investments but reduce operating costs by upwards of 60%. This article found that firms buffering strategies have been more successful than bridging activities. By examining various communities around PGM firms, it has shown how royalties, CSR, and local procurement have largely been futile efforts to improved shared-value outcomes due to challenges such as corruption and maladministration, short-term projects that are insular and tied to adverse rebound effects upon termination, and limited skills to drive higher value-added procurement. This suggests that more attention should be given to how bridging activities can be strengthened to ensure that firms can keep their social license to operate, as a loss would lead to a halt in all business activities. The findings of this research indicate that a paradigm shift may be required, such that “traditional bridging activities” should be viewed central to protecting core business activities. Royalties, CSR, and local procurement currently operate in the periphery, rather than as a central part of firms’ business strategies.

## Footnotes

1 An article in the Financial Times highlighted this, noting that “mining companies are generating so much cash at the moment due to high metals prices that it sometimes appears hard for them to know how to spend it.”

2 S&P Market Intelligence Profile

3 “Platinum prices hit by Volkswagen scandal” published on April 23, 2015 in the Financial Times.

4 Platinum dropped as low as $618 per ounce in 2020.

5 As assessed through companies’ annual reports which indicate quantities of each metal extracted.

6 HS codes 843069 (machinery; moving/grading/levelling/scraping/excavating/extracting machinery, for earth/mins./ores (excl. of 8430.10–8430.49), other than self-propelled) and 843050 (machinery; for handling earth, minerals or ores, self-propelled, n.e.c. in heading no. 8430)

7 Interviews with Anglo Platinum staff
